# Spontaneous patterning method utilizing transformation of UV-curable emulsion

**DOI:** 10.1038/s41598-022-07525-5

**Published:** 2022-03-04

**Authors:** Yoshimi Inaba, Hideo Asama

**Affiliations:** Toppan Technical Research Institute, TOPPAN Inc., Sugito, Saitama 345-8508 Japan

**Keywords:** Chemistry, Engineering, Materials science

## Abstract

A self-organizing structure is important for imparting functions and simplifying the manufacturing process. The development of spontaneous structures with a roll-to-roll process capability is a challenging task. We propose a novel patterning method utilizing the destruction of the emulsion structure. An oil-in-water (O/W) UV-curable emulsion liquid film was partially exposed to UV, resulting in aggregation and immobilization of the emulsion in the medium. Emulsion droplets in the unexposed area are coalesced by removing water. Coalesced emulsion droplets expand and spontaneously permeate into the pores formed in the aggregated structure of UV-cured emulsion particles, causing an uneven structure. An uneven pattern can be formed by direct UV exposure of the liquid film and the subsequent drying process without requiring a development process.

## Introduction

This study is on the spontaneous pattern formation technology based on a bottom–up approach. The importance of spontaneous pattern formation is that not only can high function and structure be obtained, but the process can also be greatly simplified and the energy consumption reduced^1^. An overview of previous studies on forming patterns by a self-assembly technique indicates the following main methods: (a) photoinduced mass transfer^2–6^, this method is a top-down approach, but it is exemplified as one of the prior arts in that mass transfer proceeds spontaneously after being triggered by pattern exposure, (b) the phase separation of block copolymers and polymer blends^7–12^, (c) buckling wrinkle formation on the surface by applying compressive stress^13,14^, (d) honeycomb structure formation through the surface adsorption of minute water droplets^15,16^, and (e) the physical formation of grooves or the chemical modification of patterns on a substrate on which particles are accumulated^17–19^. The features of these overviewed methods are summarized as follows. The pitch of the pattern is in the range from approximately 10 nm to 500 μm, with the aim of forming a relatively small-scale pattern. For (b–e), an additional process of providing a physical or chemical guide pattern in advance is required. On the other hand, the following methods have been proposed for optical processing using UV-curable emulsions: (f) the use of a high internal phase emulsion (HIPE)^20–25^ yields articles using UV-curable high-concentration water-in-oil (W/O) emulsion compositions; (g) pattern formation by the UV exposure of a high-concentration oil-in-water (O/W) emulsion to cure oil-phase emulsion droplets^26^. These methods require a process to remove and discard the uncured resin component.

In this study, we focused on the destruction of O/W UV-curable emulsion droplets, which has not been mentioned in previous studies using UV-curable emulsions. The structural design concept is different from the conventional patterning based on multilayer film formation or three-dimensional (3D) printing. Our approach allows spontaneous uneven pattern formation without the need for a development process. The pattern pitch extends to the millimeter scale. By controlling the mass transfer of the destructed emulsion, we can generate at once a 3D structured pattern that cannot be achieved by stacking conventional layered structures. It also paves the way for structural development in which the composition continuously changes in the horizontal direction of the substrate.

## Results and discussion

### Pattern generation by transforming UV-curable emulsion

**Figure 1:**
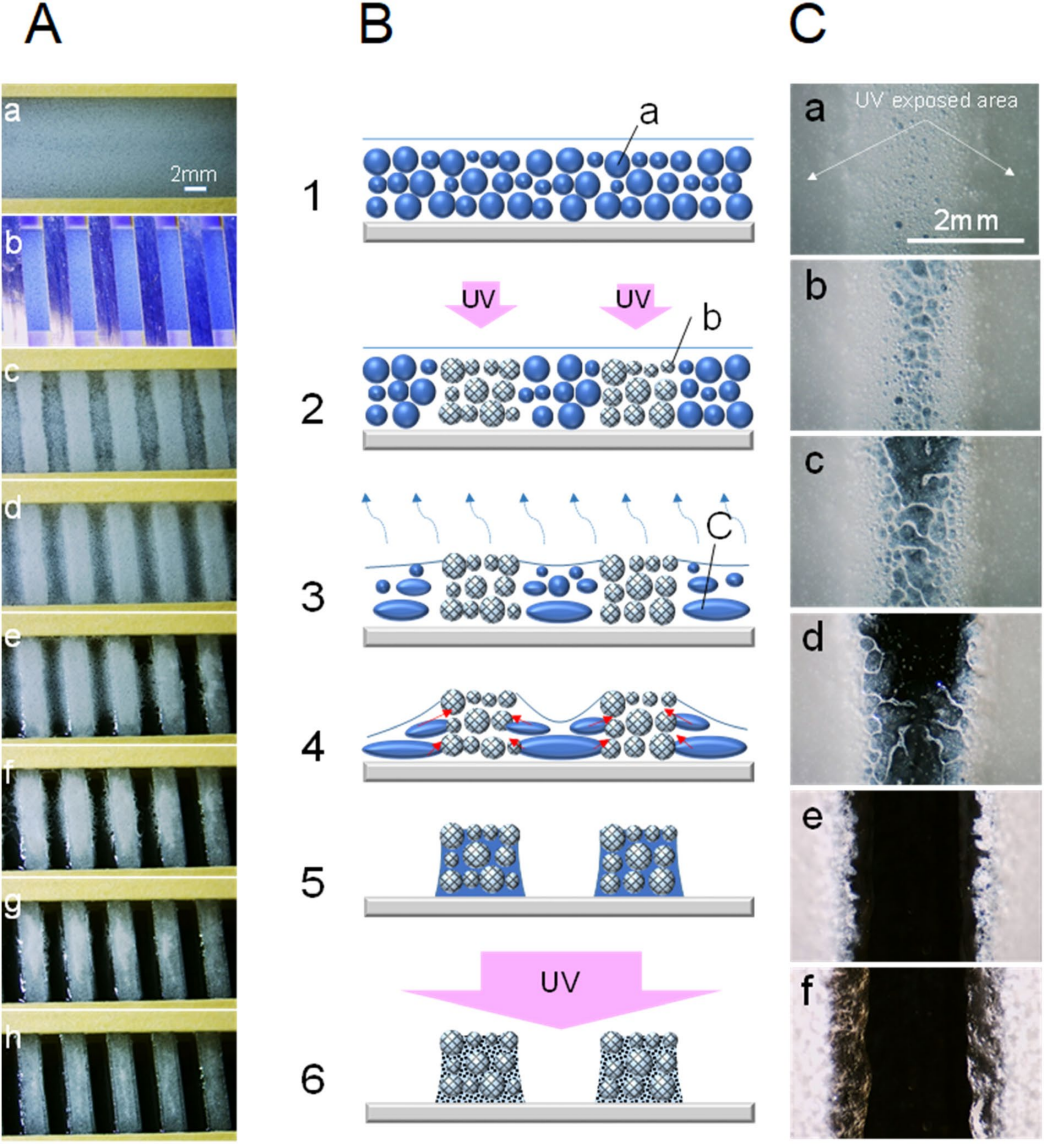
Basic principle of patterning utilizing emulsion transformation. (**A**) Stripe pattern formation by UV irradiation on the emulsion liquid film and the following dehydration process. The average particle size of the emulsion was 87.3 μm. The thickness of the emulsion layer was set to 375 μm. A 1.0-mm-thick spacer was placed between the liquid reservoir cell (refer to “Materials and methods” section; Special preparation, and Supplementary Information; Fig. S2) and the photomask. Line/space (L/S) sizes of the photomask are 2 mm/2 mm. UV illuminance of 4.6 mW/cm^2^ was applied for 8 s to the emulsion liquid surface through the photomask, and the total UV exposure was 36.8 mJ/cm^2^ (λpeak=365 nm). The UV-exposed emulsion layer was dried at room temperature (23 ℃ 57%RH) for 45 min. Finally, the entire surface of the patterned ET film was exposed to UV at a total of 183 mJ/cm^2^ as a fixing treatment. The images were cropped from a microscope video. (**a**) A liquid reservoir cell was filled with emulsion. (**b**) The cell was exposed to UV from above the mask. (**c**) Immediately after UV exposure, the mask was removed. (**d**) After 22 min of continuous drying. (**e–g**) After 30, 33, and 38 min of drying respectively. (**h**) 45 min after drying and fixing treatment by UV exposure at 183 mJ/cm^2^. (**B**) Schematic illustration of the emulsion transformation process for patterning. (**1**) Emulsion liquid film on substrate. (**2**) Pattern formation by UV irradiation. (**3**) Removal of dispersion medium by drying. (**4**) Continued drying. (**5**) End of drying. (**6**) UV exposure of entire surface. (a) UV-curable emulsion droplets. (b) UV-cured emulsion droplets. (c) Coalesced emulsion droplets. (**C**) Micrographs of the stripe pattern formed by UV exposure of emulsion taken from back of liquid reservoir cell during dehydration period. The average particle size of the emulsion was 14.9 μm. The L/S sizes of the photomask are 2 mm/2 mm. The total UV exposure was 36.8 mJ/cm^2^. The UV-exposed emulsion was dried at room temperature (25 ℃ 46%RH) for 50 min. (**a**) After 5 minutes of drying. (**b–f**) After 7, 9, 12, 25, and 50 min of drying, respectively.

In the fundamental process, an uneven (concavo-convex) pattern is formed spontaneously by UV exposure through a photomask onto the UV-curable O/W emulsion (Fig. S1) liquid film, followed by drying off the water at room temperature (Fig. 1A). It can be seen that the emulsions in the exposed area had already formed an aggregation pattern immediately after UV exposure. The emulsion droplets containing trimethylolpropane triacrylate (TMPTA) as a functional component (Table S1) in the unexposed area coalesce as the drying progresses, and the droplet size increases. As the drying progresses further, the emulsion droplets break down completely and return to a homogeneous oil (TMPTA) phase. At the same time, the homogeneous oil phase permeates into the pores formed in the UV-cured particle-aggregate layer in the UV-exposed area. After 45 min, the glass slide surface was completely visible in the unexposed area, and pattern formation was almost completed. The method is based on the idea of curing and the destruction of the emulsion form (Fig. 1B). The drying process from the third to fifth steps corresponds to the development process in a conventional lithography technology. Only the UV exposure and drying processes are required for pattern formation, and the development process for removing and discarding residues is not required. In the final step, the pattern is fixed by curing the oligomer (TMPTA) solution that has permeated between the particles cured by the UV exposure of the entire film surface.

An example of a micrograph taken from the back of the liquid reservoir cell is shown in Fig. 1C. It can be clearly seen that the emulsion droplets in the unexposed area are absorbed by the cured particle-aggregate layer in the exposed area while coalescing and breaking as the drying progresses. The pattern formation can be accelerated by increasing the drying temperature. It took about 45 min to form the pattern when dried at room temperature, but the pattern formation was completed in about 5 min when dried by heating at 81 °C (Fig. S2, Movie S1). At higher temperatures, the adsorption of the emulsifier at the surface of droplets decreases with temperature, causing the acceleration of the destruction^27^ of the emulsion. In addition, higher temperatures decrease the viscosity of TMPTA that has returned to the homogeneous phase and hence increase its penetration rate into the cured particle-aggregate layer. This process utilizes the immobilization of the generated droplet structure and the mass transfer associated with droplet destruction within the same medium, and takes advantage of changes related to the properties and shape of the emulsion droplet. We named this method the “emulsion transformation method for patterning” (ET method), and the obtained patterned film is abbreviated as ET film. In this method, a pattern can be formed by using a wide range of UV-curable acrylate and methacrylate oligomers and monomers having urethane, acrylic, epoxy structures or the like, or a blended composition thereof. A composition that can adjust a wide range of physical and chemical performance can be assembled.

### Quantitative analysis of pattern formation behavior

**Figure 2:**
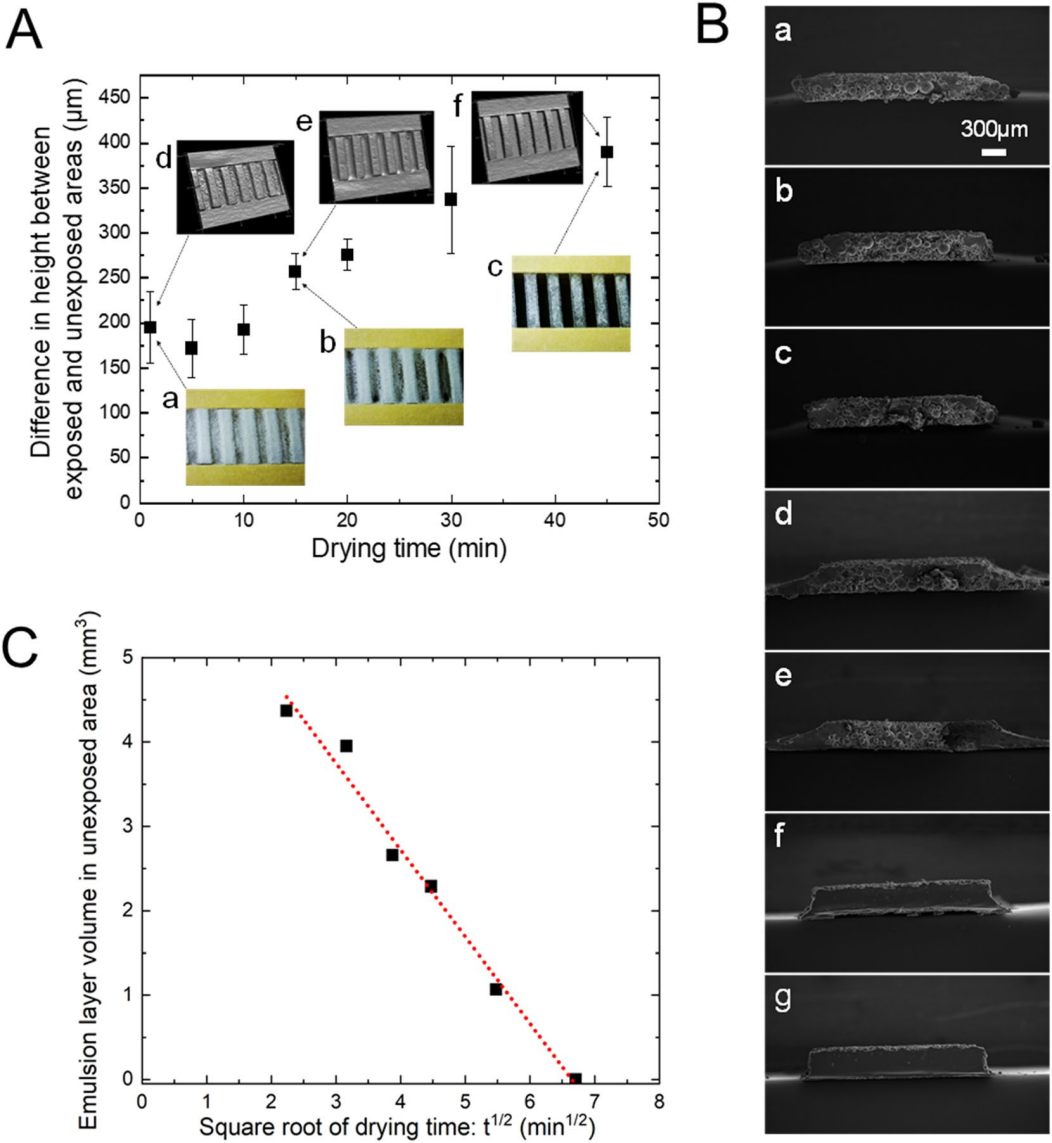
Quantitative analysis of ET method. (**A**) Relationship between drying time after UV exposure and difference in height between UV-exposed and unexposed regions. The average particle size of the emulsion was 87.3 μm. The L/S sizes of the photomask are 2 mm/2 mm. The UV illuminance was 4.6 mW/cm^2^. The total exposure was 36.8 mJ/cm^2^. The pattern formed by the UV exposure of emulsion was dried at room temperature (23 ℃ 57%RH). The height difference of the pattern was obtained by measuring the 3D shape and cutting out the cross-sectional profile. (**a**) Optical micrograph 1 min after UV exposure was completed. (**b,c**) 15 and 45 min after exposure, respectively. (**d**) 3D images 1 min after UV exposure was completed. (**e,f**) 15 and 45 min after exposure, respectively. These 3D images are stretched three times in the depth direction to emphasize the stereoscopic effect. Error bars show SD. n = 6. (**B**) Cross-sectional SEM images of the UV-exposed portion of pattern cured by entire-surface UV irradiation for fixation. (**a**) 1 min after UV exposure was finished. (**b–g**) 5, 10, 15, 20, 30 and 45 min after UV exposure, respectively. (**C**) Relationship between the square root of drying time and remaining volume of the unexposed emulsion layer. The volume *V* of the unexposed emulsion layer was estimated using the exposed pattern area (*A*: 2 mm × *B*: 10 mm) and the average height *C* (refer to (**A**)) of the emulsion deposition layer in the unexposed portion. *V* = *A* × *B* × *C*. The average height of the exposed region was used as the reference for the height of the unexposed region. R^2^ = 0.984.

Figure 2A shows the change in height difference between the exposed and unexposed areas with drying time. To quantitatively understand the process of forming the uneven pattern through drying, we adopt a total UV exposure of 36.8 mJ/cm^2^ for each duration of drying. Even the emulsion existing in the unexposed area was cured by this treatment to fix the concavo-convex structure in the liquid film. Then, the remaining water was removed by drying at room temperature for 2 h to fabricate an uneven pattern specimen. Finally, the entire surface of the specimen was exposed to UV at 138 mJ/cm^2^ to fix the pattern. Images (a–c) in Fig. 2A are optical micrographs and (d–f) are 3D shape photos. Immediately after exposure, a cured particle-aggregate layer appears. It is presumed that such rapid aggregation is mainly caused by the following mechanism: The temperature rise due to polymerization causes desorption of the surfactant adsorbed on the surface of the emulsion, the lowering of the surface potential of the particles, and the rapid destabilization of the dispersed state. After 1 min of drying, a height difference of about 200 μm was already observed. The height difference increased with the progress of drying and reached about 390 μm at 45 min. After drying for 5 min, the height difference was minimum. This behavior indicates that some of the emulsion droplets existing in the unexposed portion that is in contact with the exposed region are drawn into the natural convection that arises owing to the buoyancy generated by the temperature rise caused by polymerization at the exposed area. For this reason, the uncured emulsion droplets that have been carried by convection gather at the edge of the exposed area and sink into the original unexposed region when the convection due to heat generation stops after exposure is finished. Figure S3 shows the change in the cross-sectional profile of the film due to the drying of the residual emulsion droplets in the unexposed area after the fixing process by full UV exposure. Until 10 min after drying, a large amount of water still remains, so the coalescence of the droplets does not proceed, and a non-uniform sedimentation state is observed in the liquid film of the unexposed area. From 15 to 20 min, the coalescence with drying progresses, and the sedimentation state of the droplet reflects the meniscus shape of the liquid surface. At the same time, the coalesced droplets begins to permeate into the cured particle layer of the exposed portion. At 30 min, the glass surface of the substrate began to be clearly observed, and at 45 min, the penetration into the cured particle layer was completed and a flat surface of the glass substrate appeared.

Cross-sectional scanning electron microscopy (SEM) images of the specimen taken for each drying time are shown in Fig. 2B. When the drying time is 10 min or less, much water still remains in the emulsion film. At this stage, the coalescence of the emulsion droplets in the unexposed areas has not progressed sufficiently. Therefore, the cured and dried film is composed of only particle deposits, and the film is brittle and thus collapses during the preparation of a cross-sectional specimen. For this reason, in images (a–c), the particle deposition layer in the unexposed portion is missing. When the drying time is more than 10 min, the emulsion droplets begin to coalesce sufficiently. The coalesced TMPTA droplets permeate between the particles, fill the gaps, and adhere to each other, so that the particle layer in the unexposed portion resists collapse. In photos (d–g), the coalesced emulsion droplets permeate through the particle-aggregate layer in the exposed portion and form a convex pattern with edges that can be clearly seen. After 45 min of drying, no voids exist inside the UV-exposed pattern layer.

According to the Lucas–Washburn Eq. (1)^28,29^, the driving force for the coalesced emulsion droplets penetrating into the voids of the cured particle-aggregate layer can be predicted to be capillary force.

1 $${L}_{p}=\sqrt{\frac{e\gamma cos\theta }{2\eta }t.}$$

Here, *L*_*p*_ is the liquid penetration depth, *e* is the capillary radius, *γ* is the surface tension of liquid, *η* is the liquid viscosity, cos*θ* is the contact angle between the liquid and the capillary surface, and *t* is the elapsed time. In this case, the volume change resulting from impregnation is proportional to the square root of time. To verify this relationship, the change in the volume of the unexposed emulsion layer over time was determined as follows. The volume of the unexposed emulsion layer was calculated using the average height data in Fig. 2A and the UV-irradiated area (10 mm × 2 mm). The initial average height of the unexposed layer is set to be the same as the average height of the exposed portion after 45 min of drying. However, as described in Fig. 2A, since the positions of the emulsion particles in the liquid film about 1 min after exposure are not yet stable, the height data 5 min after exposure were used. Figure 2C shows the relationship between the square root of the drying time and the volume change of the emulsion layer remaining in the unexposed portion. Since a linear relationship can be seen from this, it can be considered that the driving force of this penetration is capillary force.

### Example of multi-layer patterning

**Figure 3:**
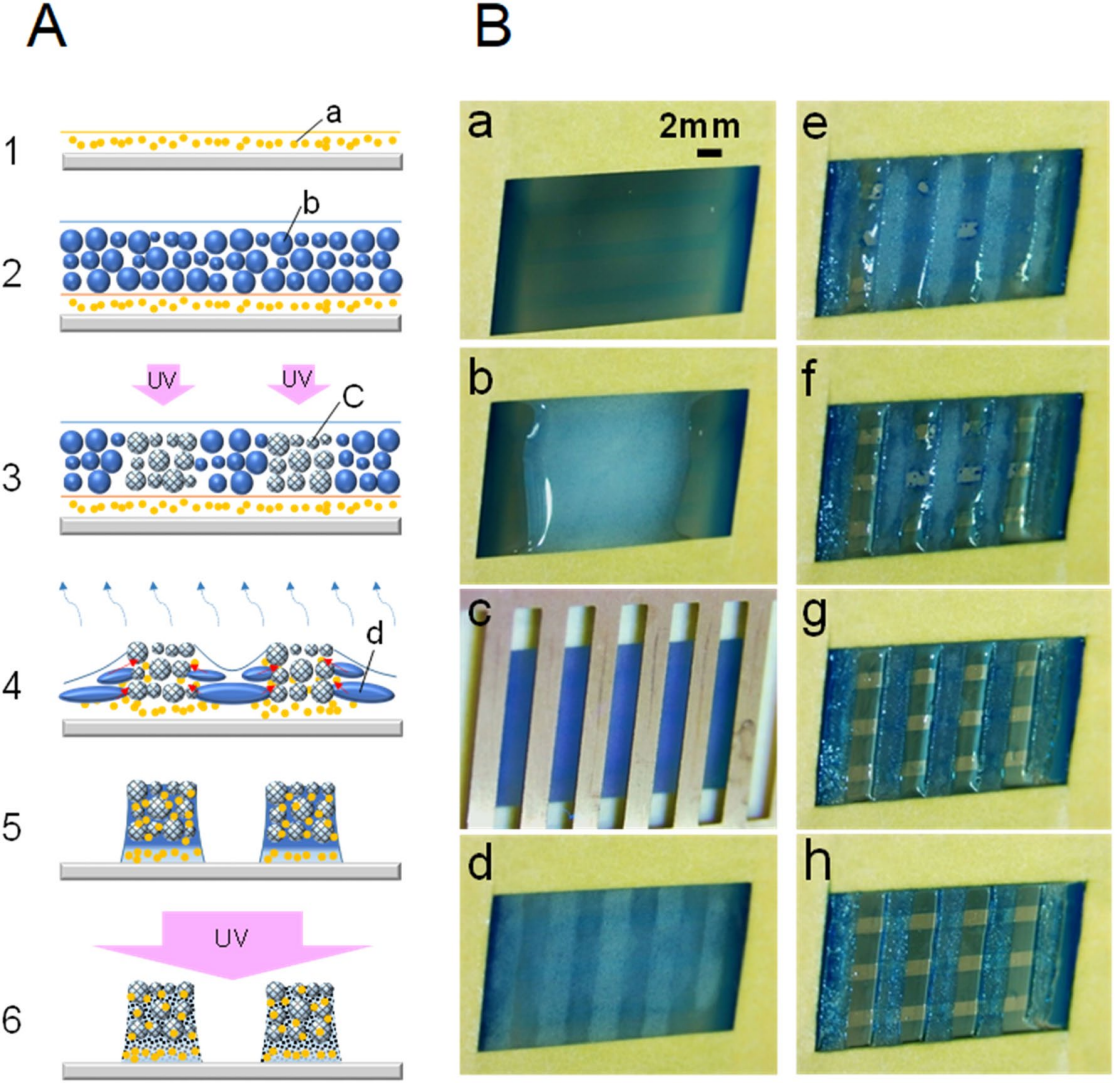
Example of two-layer patterning. (**A**) Schematic illustration of the two-layer concept of the ET method. (**1**) Formation of a resin layer on the substrate. (**2**) Emulsion liquid film on the resin layer. (**3**) UV pattern exposure of the emulsion liquid film. (**4**) Removal of dispersion medium (water) by drying. (**5**) Completion of drying. (**6**) Entire-surface UV exposure. (**a**) Resin layer. (**b**) UV-curable emulsion droplets. (**c**) UV-cured emulsion particles. (**d**) Coalesced emulsion droplets. (**B**) Example of two-layer pattern formation in which a vertical stripe pattern is formed by the ET method on a negative photosensitive resin layer provided with a lateral stripe latent image. The thickness of the resin layer was about 7 μm. (**a**) Photosensitive resin layer with the latent image formed in advance. The image was formed by contact exposure using a photomask having L/S = 2 mm/1 mm. A UV illuminance of 16.3 mW/cm^2^ was applied for 60 s, and an integrated exposure of 972 mJ/cm^2^ was performed. (**b**) Dropping and spreading of emulsion on the photosensitive resin layer. The average particle size of the emulsion was 87.3 μm. (**c**) A cell covered with a photomask was exposed to UV at 36.8 mJ/cm^2^ with an illuminance of 4.6 mW/cm^2^. (**d**) Immediately after the end of UV exposure, the photomask was removed and drying was carried out at room temperature (24 ℃ 38%RH) for 120 min. (**e–g**) After drying for 15, 20, and 30 min, respectively. (**h**) After 45 min of drying, the entire surface was fixed by exposing it to UV at 642 mJ/cm^2^.

The ET method enables the fabrication of multilayer patterns. An example of the two-layer spontaneous patterning method is shown in Fig. 3A. This is a method that positively utilizes the process of conversion from a water-based liquid to an oil-based liquid during droplet destruction by the coalescence of crude emulsions in the drying stage. In the first step, any oil-soluble resin layer will suffice as the base material on the substrate. In the second step, the emulsion is applied as the second layer onto the first layer. Since this emulsion layer is a water-based dispersion, it can be applied without dissolving the first base layer. In the third step, the emulsion layer is exposed to UV to form a partially cured particle-aggregate pattern as a kind of latent image. In the fourth and fifth steps, as a result of drying the emulsion layer, the first layer moves toward the cured particle layer of the UV-exposed region while being dissolved in the coalesced emulsion of the unexposed portion and penetrates into the voids between cured particles. This is a spontaneous development behavior against the base resin layer. If the first layer is a photosensitive resin layer on which a latent image of a pattern is recorded in advance, the underlying pattern can also appear in this spontaneous development step. In the sixth step, even if the mass transfer is completed or in progress, the pattern is fixed by the UV irradiation of the entire surface.

An example of pattern formation of a two-layer structure utilizing the ET method was demonstrated (Fig. 3B). A photosensitive resin layer formed on a glass slide was used as a base material. The composition of the photosensitive resin layer is shown in Table S2. A stripe pattern (L/S = 2 mm/1 mm) parallel to the long side of the glass slide was placed in close contact with the photosensitive resin layer and exposed. A UV-curable emulsion was applied to the photosensitive resin layer on which the latent image was formed. The emulsion was exposed to UV to form a pattern of L/S = 2 mm/2 mm in the direction perpendicular to the long side of the glass slide cell. As is clear from Fig. 3B, the unexposed part of the photosensitive resin layer was dissolved in the coalesced emulsion solution (TMPTA) during drying. While being dissolved, the emulsion solution penetrates into the cured particle-aggregate layer. It was found that a unique pattern with a two-layer structure can be produced by the spontaneous development effect. By using such a mechanism, one can create a progressive film structure design, such as a base consisting of two or more layers including a printed pattern, and the spontaneous formation of a more complex pattern can be expected.

### Forming patterns on plastic film

**Figure 4:**
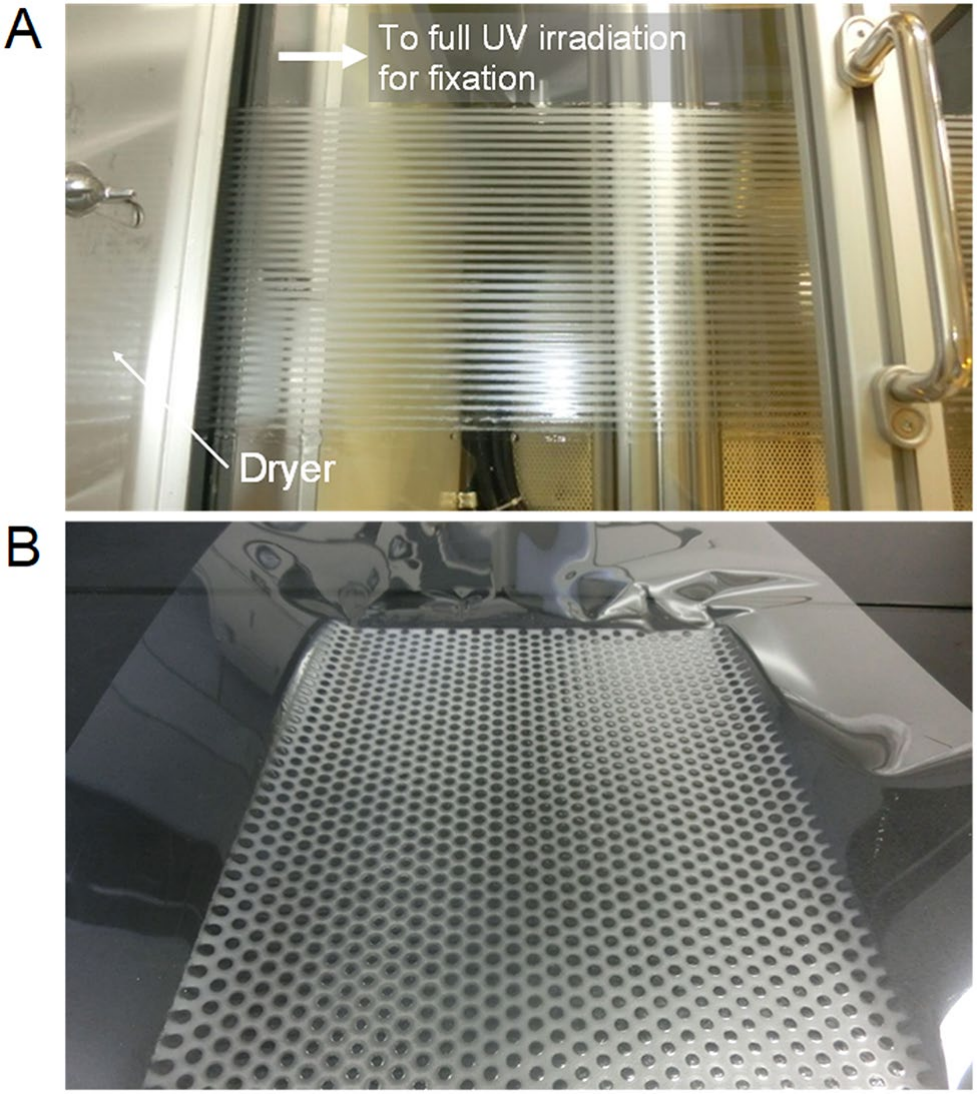
Pattern formation on plastic film. (**A**) Stripe pattern formed by roll-to-roll process using PET film with thickness of 75 μm as a substrate. The photo was taken immediately after the film passed through the drying zone. The length of the drying zone was 4 m and the temperature was set to 120 °C. The coating width was 150 mm, and the thickness of the emulsion liquid film was 100 μm. The line speed was 1.5 m/min. 24.4 mJ/cm^2^ of UV exposure was applied through the photomask with L/S = 2 mm/2 mm. The photomask was prepared by fixing a black toner pattern on a 75 μm-thick PET film with a laser printer. The distance between the photomask and the emulsion liquid surface was 5 mm. A uniform UV line exposure head consisting of an integrated crystal light fiber guide was placed in the transverse direction of the PET film. (**B**) Circular dot patterns formed by web process using a PET film with a thickness of 75 μm as a substrate. Dots, which are unexposed areas, have a diameter of 3.5 mm. The coating width is 200 mm. The thickness of the emulsion liquid film, the web process line speed, and the UV dose were the same as those in (**A**). All equipment, materials, and process geometries are the same as those in (**A**).

We demonstrated an example of producing a pattern by the ET method on a polyethyleneterephthalate (PET) film substrate (Fig. 4). Figure 4A shows an example of the continuous formation of a stripe pattern on a PET film running on a roll-to-roll coater. This photograph was taken immediately after the film passed through the dryer zone. The film was transported to the full UV irradiation zone and then rolled up. The stripe pattern was continuously exposed to UV through a slit mask. Figure 4B shows an example of a dot pattern also formed on a PET film. In this case, the dot pattern was prepared by temporarily stopping the transport of the PET film and by intermittent exposure. As can be seen from these examples, continuous winding can be performed while changing the pattern at any time, depending on the exposure system used. We believe that the ET method is a promising technology for developing industrial products.

## Conclusion

We have demonstrated that uneven patterns can be spontaneously formed by transforming the shape of emulsion droplets and mass transfer in a location-selective manner. It has been proved that it is possible to produce a large uneven pattern with a pitch of millimeter scale, which is difficult to accomplish by the previous self-assembly approach. It was shown that a complex pattern can be produced without using a relief plate by fixing and destroying the emulsified structure in the same medium. This method utilizes absorption flow due to the capillary force, and it has the advantage that the cleaning and waste liquid treatment steps associated with development can be eliminated. The absorption flow can also be utilized as a means of supplying another material to the voids of the porous pattern formed in a location-selective manner. The ET method is advantageous for forming various patterns on plastic films and can be expected to be applied industrially. ET films will provide new textures in a wide range of applications such as interior decor materials, unique patterned pressure-sensitive adhesive, and location-selective light diffusion films. Moreover, ET method can be preferably applied for mm wave antenna^30–32^ where the antenna pattern is in the range of millimeter and submillimeter. We see a huge potential for the application of our method to the pattern for metamaterial for electromagnetic waves in the band of 25 GHz or higher.

## Materials and methods

### Materials

Trimethylolpropane triacrylate (TMPTA) (Light Acrylate TMP-A, Kyoeisha Chemical Co., Ltd.) was employed as the oligomer without additional treatment. 1-Hydroxycyclohexyl phenyl ketone (Lunacure 200, DKSH Japan) and 2,4,6-trimethylbenzoyl-diphenyl-phosphine oxide (Lucirin TPO, BASF) were used as photoinitiators. An emulsifier (Sanmorin OT-70, Sanyo Chemical Industries, Ltd.) consisting of a mixture of 70% sodium dioctyl sulfosuccinate, 16% propane-1,2-diol (propylene glycol) and 14% water was used as received. Distilled water was used as the dispersion medium. Kayaset Blue N (Nippon Kayaku Co., Ltd.) was used as the blue dye for the oligomer and resin. Macromonomer AA-6 (Toagosei Co., Ltd.) was used as a solvent-soluble reactive high-molecular-weight methacrylic monomer for preparing the photopolymer. n-Butyl acetate was used as the solvent.

### Emulsification

Into a 50 mL brown vial, the following materials were put one at a time in the following order: Lunacure 200, Sanmorin OT-70, and TMP-A. The vial was rotated for 2 h to mix the materials, and then distilled water was added. Emulsification was performed using a paint shaker and manual shaking. Shaking using a paint shaker (PC1171, Asada Iron Works) was carried out for 30 s. The manual shaking involved 10 repetitions of intermittent shaking at intervals of 20 s. The rest intervals provided the time required for the emulsifier as the stabilizer to diffuse to the newly generated interface^33^. After the above treatment, the vial was further subjected to rotary stirring for 2 h. Table S1 shows the formulation of the emulsion model. The size distributions of emulsion droplets are shown in Fig. S1. The weight-average particle sizes of the emulsions prepared using the paint shaker and by manual shaking are 87.3 μm and 14.9 μm, respectively.

### Special preparation

A 1.0-mm-thick glass slide was used as a substrate for forming an emulsion liquid film. Five layers of 80-μm-thick 3 M masking tape were attached to the glass slide. Then, the central part was cut out in the shape of a rectangle of 10 mm × 20 mm or 10 mm × 30 mm to form a bank. This cut-out portion serves as a liquid reservoir cell. A liquid film simulating the coating layer was formed by dropping the emulsion into the liquid reservoir cell, so that the thickness calculated using the specific gravity of the emulsion would be 375 μm. A 1.0-mm-thick aluminum plate spacer was placed on the liquid reservoir cell surface, and a photomask was placed on the spacer. The photomask was a copper plate with a thickness of 0.25 mm. Two types of UV light source were employed. Parallel UV light equipment (UVC-2502S, superhigh-pressure mercury lamp, λ_peak_ = 365 nm, 405 nm, 436 nm; San-Ei Electric Corp.) was used in the laboratory experiments. An ultrahigh-pressure mercury lamp (UL750, Hoya Candeo Optronics Corp.) equipped with a crystal fiber light guide head was employed in roll-to-roll process patterning experiments. The photomask was removed from the liquid reservoir cell when the UV exposure of the pattern was finished, and the cell was dried. After the completion of drying, entire-surface UV exposure was applied for fixing.

### Observation and measurement

To obtain the size distribution of emulsion droplets, the weight-average diameter was measured using a particle size distribution measuring system (Microtrac MT3300EXII, Nikkiso Co., Ltd.) equipped with a liquid circulation pump (Microtrac USVR, Nikkiso Co., Ltd.). Two types of optical microscope were used to observe the emulsion droplets and patterned film. The BX51 microscopy system (Olympus Corp.) was used for still-image shooting. For video shooting, a zoom microscope (L-815, Hozan Tool IND. CO., LTD.) equipped with a USB camera (L-835, Hozan Tool IND. CO., LTD.) was used. The height difference of the pattern was obtained by measuring the shape of the uneven pattern and extracting the cross-sectional profile. Aluminum with a thickness of 800 Å was formed on the surface of the patterned specimen using vacuum vapor deposition equipment (VC-500P, JEOL Ltd.), and then, the three-dimensional (3D) shape was measured using the one-shot 3D shape measuring instrument (VR3100, Keyence Corp.). The height difference of the pattern was directly obtained from the cross-sectional profile data. A scanning electron microscopy (SEM) system (Carry Scope JCM-5100, JEOL Ltd.) was used for the direct observation of the pattern cross section.

## Supplementary Information

Supplementary Information.

Supplementary Video 1.
