# Supplementary material for: Spontaneous patterning method utilizing transformation of UV-curable emulsion
Source: Sci Rep. 2022 Mar 4;12:3607. doi: 10.1038/s41598-022-07525-5 (PMC8897442; doi:10.1038/s41598-022-07525-5)
Supplement: Supplementary file 1 — Supplementary Information. [file 41598_2022_7525_MOESM1_ESM.pdf]

Supplementary Information

Spontaneous patterning method utilizing transformation of UV-curable emulsion

Yoshimi Inaba[1,*], Hideo Asama[1]

[1]Toppan Technical Research Institute, TOPPAN Inc.; Sugito, Saitama 345-8508 Japan.

Correspondence to: yoshimi.inaba@toppan.co.jp

This PDF file includes:

Tables S1 to S2

Figures S1 to S3

Caption for Movie S1

Other Supplementary Information for this manuscript include the following:

Movie S1

Table S1. Contents of emulsion models.

Ingredients	Amount (g)
Trimethylolpropane triacrylate	7.5
1-Hydroxycyclohexyl phenyl ketone	0.375
Sanmorin OT-70[(a)]	0.259
Distilled water	9.0

[(a)] Sanmorin OT-70: mixture of 70 wt% dioctyl sodium sulfosuccinate, 16 wt% propylene glycol, and 14 wt% water.

Table S2. Photosensitive resin composition.

Ingredients	Amount (g)
Macromonomer AA-6[(a)]	7.0
Trimethylolpropane triacrylate	3.0
2,4,6-Trimethylbenzoyl-diphenyl-phosphine oxide	0.6
Kayaset blue N[(b)]	0.2
Butyl acetate	100.0

[(a)]Macromonomer AA-6: high-molecular-weight monomer with a polymerizable functional group of methyl methacrylate. This material is a powder.

[(b)]Kayaset Blue N (C.I. Solvent Blue 35): oil-soluble blue dye.

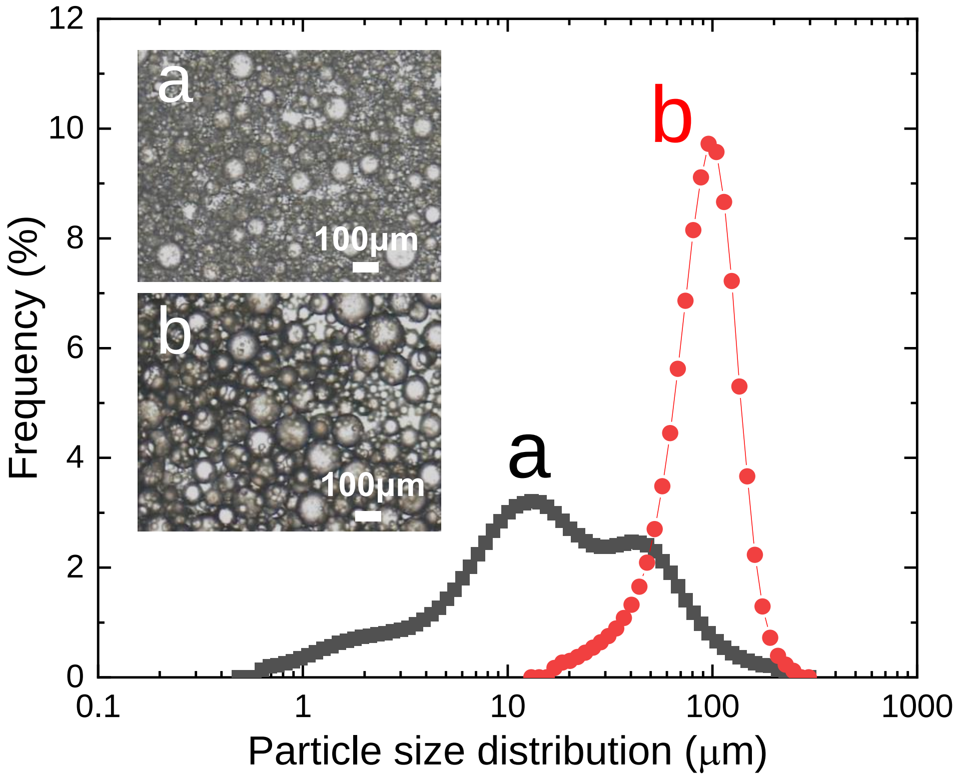

Figure S1. Particle size distribution of UV-curable crude emulsions employed in this study. (**a**) Emulsified by intermittent manual shaking. The weight-average particle size obtained was 14.9 μm. (**b**) Emulsified using a paint shaker. The weight-average particle size obtained was 87.3 μm.

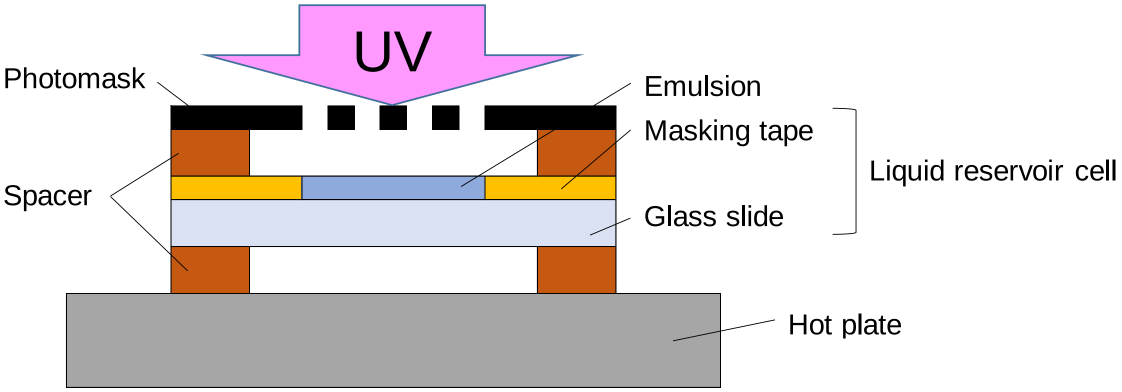

Figure S2. Layout of high-temperature drying in ET method. The distance between the hot plate and the liquid reservoir cell is 1 mm. The thickness of the masking tape layer is 0.4 mm. The distance between the surface of the masking tape and the photomask is 1 mm. The photomask thickness is 0.25 mm. L/S=2 mm/2 mm. Parallel UV light equipment was employed for uniform exposure.

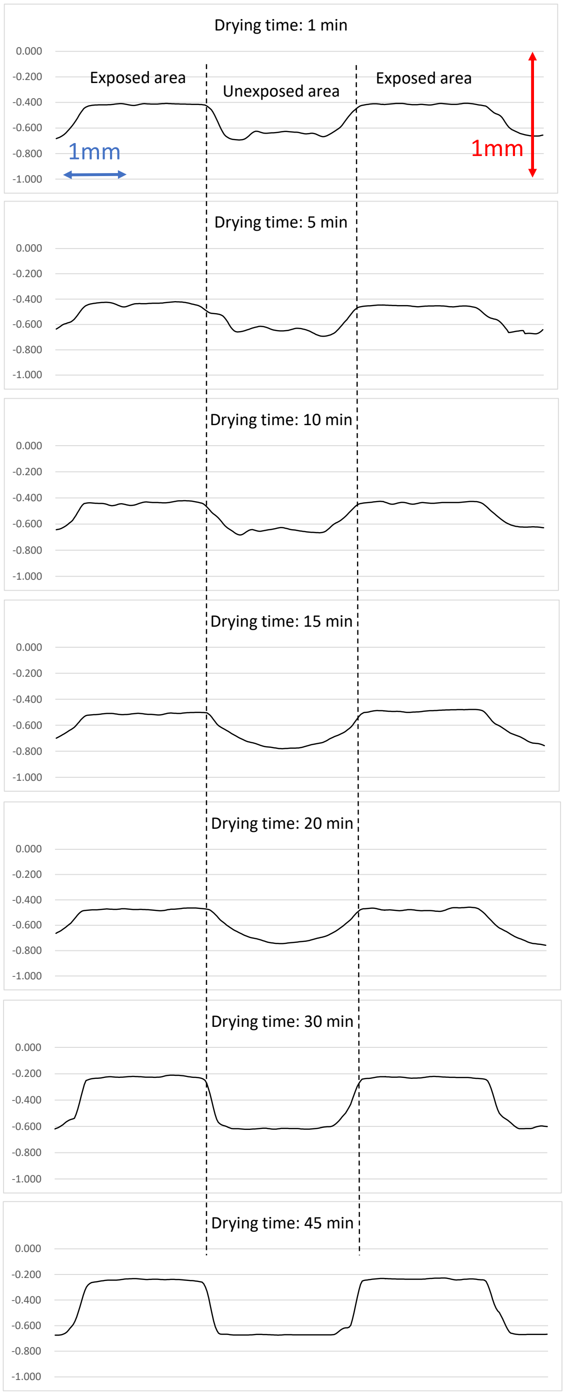

Figure S3. Room temperature drying of the ET pattern prepared on the glass slide liquid reservoir cell and the pattern cross-sectional profile after UV full exposure fixing. The vertical axis is stretched twice as much as the horizontal axis to emphasize its dimension.

Movie S1. High-temperature drying in ET method. UV with an illuminance of 4.6 mW/cm^2 was exposed for 8 s through a photomask with L/S= 2 mm/2 mm at room temperature. Immediately after the end of UV exposure, the heating of the hot plate set to 100 ℃ was started, and 100 ℃ was reached about 1 min later. The surface temperature of the glass slide cell reached 81 °C and was stable. Drying was completed in about 5 min. A pattern similar to that in Fig. 1A was formed.
